# Clinical and Demographic Characteristics of Oral Sarcoidosis: A Systematic Review of Case Reports and Case Series

**DOI:** 10.3390/jcm14197006

**Published:** 2025-10-03

**Authors:** Mohamed Jaber, Nadin Abouseif, Mawada Abdelmagied, Essra Mohamed El-Ameen

**Affiliations:** 1 Department of Clinical Sciences, College of Dentistry, Ajman University, Ajman P.O. Box 346, United Arab Emirates; m.abdelmagied@ajman.ac.ae; 2 Center of Medical and Bio Allied Health Sciences Research, Ajman University, Ajman P.O. Box 346, United Arab Emirates; essrajaber@gmail.com; 3 College of Medicine, Ajman University, Ajman P.O. Box 346, United Arab Emirates

**Keywords:** oral Sarcoidosis, bone involvement, gingival hyperplasia granulomatous disease, oral manifestations, jawbone Sarcoidosis, oral mucosal lesions

## Abstract

**Background/Objectives**: Sarcoidosis is a granulomatous disorder of unknown etiology that can affect multiple organs, including the oral cavity. This study aimed to compare the clinical and demographic characteristics of sarcoidosis cases with and without bone involvement in the jaw. **Methods**: A systematic review of the case reports and case series of sarcoidosis in the oral cavity between 1943 to 2024 were analyzed. Variables assessed included age, sex, presenting symptoms, duration of symptoms, diagnosis methodology, treatment approaches, and outcomes. **Results**: A total of 59 studies reporting 77 patients were included, with a mean age of 43.3 yrs. Female predominance was noted in both, bone-involved (61.5%) and non-bone-involvement cases (72.5%). Patients with bone involvement often presented with localized symptoms such as loose teeth (34.6%), bone loss (69.2%), and nasal obstruction (15.4%), whereas non-bone-involvement cases frequently exhibited soft tissue manifestations, like swelling (38%) and bleeding (14%). Treatment typically involved surgical intervention and steroid therapy in both groups, with favorable outcomes achieved in most cases. **Conclusions**: This systematic review presents the most extensive analysis of oral sarcoidosis. Oral sarcoidosis presents as two distinct clinical entities based on bone involvement. Soft tissue lesions often serve as an early diagnostic clue for systemic disease, while bony manifestations suggest a later, more destructive complication. Recognizing this dichotomy is crucial for dentists and clinicians to ensure timely diagnosis and appropriate referral, and this underscores the oral cavity’s critical role as an indicator of systemic illness and mandates a multidisciplinary management strategy.

## 1. Introduction

Sarcoidosis is a multisystem granulomatous disorder of unknown etiology, characterized by the formation of non-caseating granulomas. While primarily affecting the lungs and lymphatic system, it can involve virtually any organ, including the oral cavity. Oral manifestations are rare, reported in only 1–2% of cases, but they present a significant diagnostic challenge for dental practitioners [1,2].

These orofacial presentations—which can include mucosal nodules, gingival hyperplasia, swelling, and even destructive jawbone lesions—are often non-specific and easily mistaken for more common conditions like periodontal disease, foreign body reactions, or other granulomatous infections [3,4]. This frequently leads to delayed diagnosis. Furthermore, oral lesions can be the initial sign of systemic sarcoidosis or represent a localized complication in patients with established disease, creating a complex clinical scenario [5]. A key unresolved question is whether sarcoidosis involving the jawbone constitutes a distinct, more aggressive clinical phenotype compared to disease confined to the soft tissues.

Despite its clinical importance, the literature on oral sarcoidosis is limited to case reports and small series, making it difficult to synthesize a clear understanding of its demographic patterns, clinical behavior, and optimal management. A detailed analysis comparing cases with and without bone involvement is lacking.

Therefore, the aim of this systematic review is to comprehensively analyze and compare the clinical and demographic characteristics of sarcoidosis patients with and without jawbone involvement. By synthesizing evidence from published cases, this study seeks to clarify these distinct presentations, highlight key diagnostic clues, and inform therapeutic considerations to improve patient outcomes in dental practice.

## 2. Materials and Methods

### 2.1. Protocol & Registration

This systematic review was carried out in accordance with the guidelines of the Preferred Reporting Items for Systematic Reviews and Meta-Analyses (PRISMA) statement [6]. This review was registered with PROSPERO [ID: CRD42024549847].

### 2.2. Search Strategy and Information Sources

A thorough literature search was carried out across PubMed, Scopus, Embase, ScienceDirect, EBSCO, and Google Scholar to identify relevant studies. The search utilized targeted phrases in the advanced search builder, using Boolean operators ‘OR’ and ‘AND” to broaden the search. Search string used in PubMed: (((“mouth”[MeSH Terms] OR “mouth”[All Fields] OR “oral”[All Fields]) AND (“sarcoidosis”[MeSH Terms] OR “sarcoidosis”[All Fields] OR “sarcoidoses”[All Fields])) OR ((“intraoral”[All Fields] OR “intraorally”[All Fields]) AND (“sarcoidosis”[MeSH Terms] OR “sarcoidosis”[All Fields] OR “sarcoidoses”[All Fields]))) AND (“oral manifestations”[MeSH Terms] OR (“oral”[All Fields] AND “manifestations”[All Fields]) OR “oral manifestations”[All Fields] OR (“oral”[All Fields] AND “manifestation”[All Fields]) OR “oral manifestation”[All Fields]).

Equivalent search strategies were adapted for Scopus, Embase, EBSCO, ScienceDirect, and Google Scholar. Filters Applied: Language: English, Article types: Case reports, case series, and population: Human, with no time limitations. The final search performed was in March 2025.

Records were reviewed by title, abstract, and full text by two independent reviewers (N.A. and M.A.). The selected articles were read in full to assess their eligibility. A list of excluded studies was maintained and updated periodically to prevent selection bias between the two independent reviewers. When the reviewers had differing views, they resolved the issue through discussion, and a third senior reviewer (M.J.) was consulted if consensus could not be reached.

### 2.3. Eligibility

The studies included in this review met all the predefined criteria specified by the PECOS framework [7] (“Population,” “Exposure,” “Comparison,” “Outcomes,” and “Study design”) presented in Table 1.

### 2.4. Data Extraction

Prior to synthesis, the extracted data were standardized to ensure consistency across the included case reports and case series. Two independent reviewers extracted required information from included articles. Data extracted included author(s) and year of publication, sample data: age & gender, location, initial symptoms, time to presentation, diagnosis of sarcoidosis (new/pre-existing), treatment, and outcome (unclear, resolved, spontaneous remission, no change). The data were presented in two tables, one for sarcoidosis with bone involvement and the second of sarcoidosis in soft tissue only (Tables S1 and S2) [8,9,10,11,12,13,14,15,16,17,18,19,20,21,22,23,24,25,26,27,28,29,30,31,32,33,34,35,36,37,38,39,40,41,42,43,44,45,46,47,48,49,50,51,52,53,54,55,56,57,58,59,60,61,62,63,64,65,66].

### 2.5. Study Quality Assessment

The critical appraisal of case reports and case series was assessed according to The Joanna Briggs Institute Critical Appraisal tools for JBI Systematic Reviews issued by the Faculty of Health and Medical Sciences at the University of Adelaide, South Australia [67]. Two independent investigators completed these questionnaires, and any disagreements were resolved through discussion between them (Table S3).

### 2.6. Statistical Analysis

Given the nature of the included studies (heterogeneous case reports), a meta-analysis was not feasible. Data were synthesized narratively and descriptively. Categorical variables were summarized as frequencies and percentages, and continuous variables as means with standard deviations or medians with ranges, as appropriate (Table 2).

Recognizing the inherent limitations of applying inferential statistics to aggregated case data, which does not constitute a representative population sample, comparative analyses (Chi-square/Fisher’s exact tests) were performed cautiously and for exploratory purposes only. Their purpose was to identify potential associations and patterns within this collected cohort to inform future research questions, not to provide definitive statistical conclusions. A *p*-value of <0.05 was considered statistically significant for these exploratory analyses. All analyses were performed using SPSS version 28.

## 3. Results

### 3.1. Literature Search

**Figure 1:**
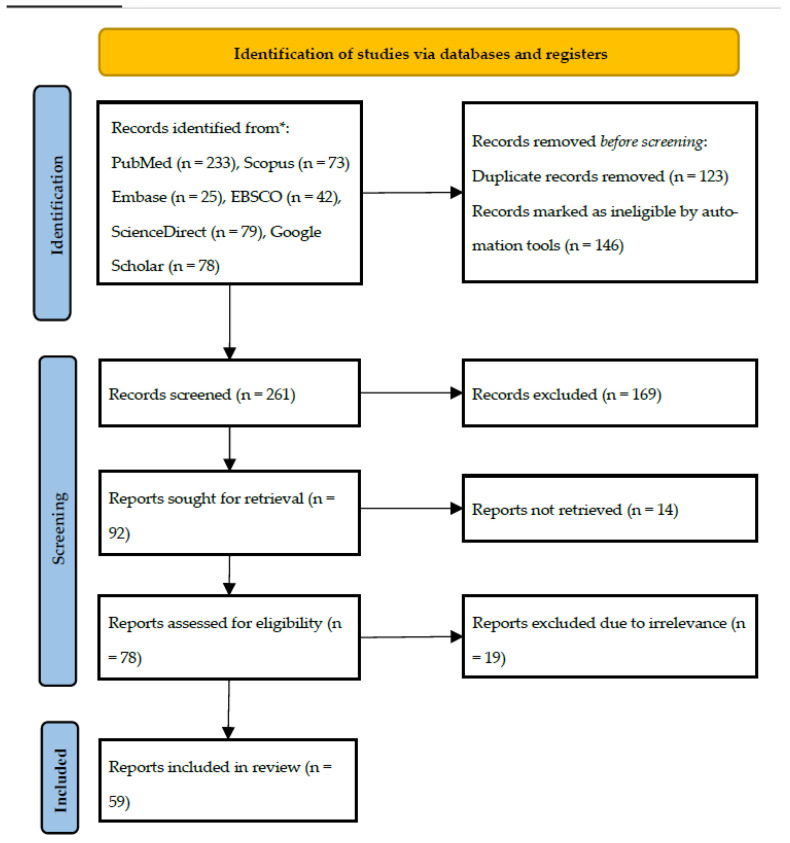
PRISMA flow diagram.

The database search resulted in a total of 530 articles. After filtering for articles published of sarcoidosis in humans in English, and removing duplicates, 261 articles remained to be screened. After reviewing the abstracts, 169 were excluded. The full text of the remaining 92 articles were retrieved for assessment of eligibility. Of those 92 articles, 14 were not retrieved, leaving 78 articles to be further evaluated according to our inclusion and exclusion criteria and to be critically appraised. Finally, a total of 59 articles were included in this systematic review following the search criteria (Figure 1).

### 3.2. Study Quality Assessment

Included studies were assessed using The Joanna Briggs Institute Critical Appraisal tools for JBI systematic reviews [67]. The tool focuses on sufficient demographics, history, presentation, diagnosis, and proper intervention. Studies scoring 7–8 were deemed as having a “high quality,” scoring 4–6 was “moderate quality,” and scores of ≤3 were “poor quality.”. Of the 59 articles, 40 scored high quality and 19 scored moderate. The single-case design of most reports limits the robustness of the evidence, warranting cautious interpretation of the results. Moderate scores were due to a lack of data on some criteria (Table S3).

Criteria:

1. Patient demographics clearly described
2. Patient history and presenting symptoms adequately described
3. Current clinical condition clearly described
4. Diagnostic tests or methods and findings clearly reported
5. Intervention or treatment procedure clearly described
6. Post-intervention condition described
7. Adverse events or unanticipated outcomes reported
8. Case report provides takeaway lessons

### 3.3. Study Characteristics

Fifty-nine articles that presented 77 patients met the inclusion criteria. The 59 articles were case reports and case series published between 1943–2024. Of the 59 articles, 57 were case reports, presenting 1–3 cases per article, while 2 were case series of oral sarcoidosis. The sample size was 77 patients diagnosed with oral sarcoidosis, total new cases diagnosed was *n* = 48 and remaining 29 cases already had history of systemic sarcoidosis. Patients age ranged between 14–85 years, and females were more affected than males, with a ratio of 2.2:1.

#### 3.3.1. Sarcoidosis with Jaw Bones Involvement (Table S1)

The average (SD) age of patients at presentation was 42.3 years (13.7), with the youngest patient being 22 years and the oldest 74 years. Female patients make up the majority (61.5%). The most commonly involved location in bone-involved sarcoidosis was found to be the mandible (12 cases), followed by maxilla (10 cases), and less frequently it would be generalized (4 cases). Common symptoms include bone loss, loose teeth, jaw pain, nasal obstruction, and gingival recession. In some cases, symptoms are absent and detected during routine dental check-ups. The onset varies from 1 month to over a decade, suggesting insidious progression in many cases. Diagnoses often follow imaging and biopsies taken from the bone affected to confirm diagnosis of bone-involved sarcoidosis. Most of the cases were diagnosed in patients with already pre-existing sarcoidosis (53.8%), while in the remaining cases the presented symptoms lead to the new diagnosis of sarcoidosis in these patients. Treatments frequently involve surgical intervention, alone or combined with steroids. Outcomes range from resolved to unclear, with one case worsening. Prognosis varies significantly, with complex cases requiring rigorous management.

#### 3.3.2. Sarcoidosis Without Jaw Bones Involvement (Table S2)

The average age is slightly older at 44.3 years (range 14–85). Females dominate (72.5%), with males representing only 27.5%. Common locations include the gingiva, lips, and tongue. Symptoms include localized swellings or nodules, ulcerations, erythema, typically without pain, and in some cases mild bone loss. Some cases remain asymptomatic, while others present more pronounced discomfort, such as burning sensations or bleeding. The timeline is similarly diverse, ranging from days to years, reflecting acute and chronic presentations. Diagnoses rely on clinical and histopathological examinations, performed on specimens obtained from the soft tissues. Treatments often involve steroids (topical or systemic), with surgical options reserved for severe cases. Most outcomes are favorable, with spontaneous remissions observed in some. Prognosis is generally favorable, with minimal structural damage and conservative management sufficing in many cases.

#### 3.3.3. Comparison Between Sarcoidosis with and Without Bone Involvement (Table 2)

Sarcoidosis involving bone affects both genders relatively equally, while sarcoidosis without bone involvement is predominantly found in females, with a ratio of 2.64:1. Patients with bone involvement are mostly adults, with ages spanning from 22 to 74, whereas soft tissue-only cases range from 14 to 85 years. Statistically, bone involvement was significantly associated with structural changes such as bone loss (69.2% vs. 9.8%, *p* < 0.001), tooth mobility (34.6% vs. 2.0%, *p* < 0.001), and nasal obstruction (15.4% vs. 0%, *p* = 0.011). In contrast, non-bone-involvement cases more frequently exhibited swelling (37.3% vs. 15.4%, *p* = 0.04) and bleeding (13.7% vs. 0%, *p* = 0.048). Additionally, a statistically significant difference was observed between the two groups regarding diagnosis, with bone-involved sarcoidosis more often occurring in patients with a pre-existing diagnosis (53.8% vs. 29.4%, *p* = 0.033). Clinically, this suggests that osseous lesions may represent a later manifestation of systemic sarcoidosis, whereas soft tissue lesions are more likely to serve as the first diagnostic clue. There was no statistical significance in terms of location, treatment, and outcomes of cases between both groups.

## 4. Discussion

Sarcoidosis is a systemic granulomatous disease; the oral manifestations of sarcoidosis can appear in the oral cavity as an initial presentation of the disease and at any time during its development [68,69,70]. Thus, it is essential for dentists to understand and recognize the oral manifestations of the disease in order to prevent the misdiagnosis of oral lesions. In addition, multiple factors must be considered if surgery or biopsy procedures are planned for oral care.

Oral sarcoidosis is considered uncommon and can present in association with or independently from the pulmonary disease [55,71]. Lesions in the oral cavity are often asymptomatic, and the incidence of a misdiagnosis is very high because of the low degree of awareness by dentists of the different aspects of the disease [71]. Moreover, dental care should be modified due to existing treatments.

This study provides valuable insights into the distinct clinical and demographic profiles of sarcoidosis, this thorough comparison contextualizes the findings within the broader literature, offering valuable insights into the management and prognosis of sarcoidosis in the oral cavity. The variability in outcomes underscores the importance of early diagnosis and comprehensive management strategies. Non-invasive diagnostic methods and standardized treatment protocols could improve outcomes.

The low incidence of mandibular and maxillary sarcoidosis in this study contrasts with studies by Betten and Koppang, where osseous involvement was frequently underestimated due to subtle radiological changes [11]. Sarcoidosis involving bone tends to have a more significant impact on dental structures and requires extensive management, while non-bone sarcoidosis often presents in soft tissues and is managed conservatively with good outcomes. Both types demand careful monitoring, though bone involvement necessitates a more rigorous approach due to potential structural complications. This study sheds light on the patterns of sarcoidosis involving bone of the jaw and sarcoidosis without bone involvement, revealing distinct demographic, clinical, and therapeutic characteristics.

The strong female predominance (2.2:1) observed in our cohort aligns with the well-established epidemiology of systemic sarcoidosis, which demonstrates a higher prevalence in women, particularly in African American populations [72,73]. This consistency suggests that oral manifestations reflect the same underlying demographic risk factors as the systemic disease.

The most critical finding is the stark contrast between bone-involved and soft-tissue involved sarcoidosis. Bone-involved sarcoidosis presented as a destructive osteolytic process, characterized by high rates of bone loss (69.2%, *p* ≤ 0.001) and tooth mobility (34.6%, *p* ≤ 0.001). Crucially, over half of these cases (53.8%) occurred in patients with a known systemic diagnosis. This aligns with the established understanding of osseous sarcoidosis elsewhere in the body, which is often a later-stage, chronic manifestation and is frequently asymptomatic until significant structural damage has occurred [5,31]. Our findings confirm that the jawbone is not exempt from this pattern.

Conversely, soft-tissue oral sarcoidosis (nodules, swelling, hyperplasia, bleeding) was significantly more likely to be the initial presenting sign of systemic disease (70.6%). This finding has profound diagnostic implications. It positions the dental professional on the front line of systemic disease detection. A patient presenting with persistent, unexplained gingival swelling or a mucosal nodule—lesions that are often biopsied by dentists to rule out malignancy or localized pathology—may in fact be presenting the first clue of a multisystem disorder.

For patients without a systemic diagnosis: Unexplained soft tissue lesions (e.g., swelling, nodules, hyperplasia) should expand the differential diagnosis to include sarcoidosis. A biopsy revealing non-caseating granulomas must prompt referral to a physician for further investigation, including chest imaging (e.g., radiograph or CT to identify bilateral hilar lymphadenopathy) and serological testing (e.g., serum ACE levels, though non-specific) [74,75,76].

For patients with a known systemic diagnosis, the development of new symptoms like tooth mobility or radiographic evidence of unexplained bone loss should raise strong suspicion for jawbone involvement, necessitating advanced imaging (e.g., CBCT or CT) and biopsy to confirm sarcoidosis activity and rule out other causes of osteolysis.

In both scenarios, close collaboration with physicians (pulmonologists, rheumatologists) is essential for comprehensive patient management.

Moreover, systemic sarcoidosis (mostly cardiac or pulmonary) was diagnosed following oral presentation in 73% of our newly diagnosed cases, while the remaining 27% only had oral sarcoidosis and no systemic manifestations. This highlights the role of oral health professionals in identifying soft tissue changes that may prompt further systemic evaluation, while also emphasizing the need for ongoing monitoring of patients with known sarcoidosis for possible later bone involvement

The treatment approach for oral sarcoidosis based on the clinical phenotype and extent of disease. For localized, minor, asymptomatic, or isolated oral soft-tissue lesions (e.g., small nodules, focal gingival enlargement), first-line management often involves local therapies. This includes intralesional corticosteroid injections (e.g., triamcinolone acetonide) or potent topical corticosteroids, which can effectively reduce granulomatous inflammation without systemic side effects [30,60].

However, for more extensive soft-tissue involvement, symptomatic lesions, or destructive jawbone disease, systemic therapy is typically required. Oral corticosteroids (e.g., prednisone) are the first-line systemic agents to control the underlying granulomatous activity [16,32]. Bone-involved disease frequently necessitates a combined approach: systemic corticosteroids to halt disease progression, coupled with surgical intervention (curettage, debridement, or resection) to address structural damage, obtain definitive histopathological diagnosis, and alleviate symptoms like tooth mobility [11,30].

In cases of corticosteroid resistance or for long-term steroid-sparing management, second-line immunosuppressive agents such as methotrexate or biological therapies like anti-TNF-α agents (e.g., infliximab) may be employed, particularly for patients with concurrent severe systemic involvement [31,36,37].

While outcomes were generally favorable in both groups, the potential for recurrence and chronicity, especially in bone-involved cases, necessitates long-term, multidisciplinary follow-up.

This study highlights the distinct patterns of sarcoidosis with and without bone involvement, emphasizing the need for tailored diagnostic and management strategies. Both forms of the condition demand nuanced approaches to optimize patient outcomes. Furthermore, this study enhances the understanding of sarcoidosis in the oral cavity, emphasizing the need for: targeted diagnostic approaches for bone versus non-bone involvement, awareness among clinicians to reduce diagnostic delays and multidisciplinary management strategies for complex cases. Future research should explore the underlying immunological and genetic mechanisms influencing the localization and progression of sarcoidosis.

## 5. Limitations

The conclusions of this review are constrained by the inherent limitations of its source data—case reports and series. This introduces a potential for publication and selection bias, as more severe or unusual cases are more likely to be published. The application of inferential statistics is exploratory, and the findings require validation in larger, prospective cohort studies.

## 6. Conclusions

This systematic review presents the most extensive analysis of oral sarcoidosis. Oral sarcoidosis presents as two distinct clinical entities based on bone involvement. Soft tissue lesions often serve as an early diagnostic clue for systemic disease, while bony manifestations suggest a later, more destructive complication. Recognizing this dichotomy is crucial for dentists and clinicians to ensure timely diagnosis and appropriate referral, and this underscores the oral cavity’s critical role as an indicator of systemic illness and mandates a multidisciplinary management strategy.

## Figures and Tables

**Table 1:** PECOS inclusion and exclusion criteria.

Parameter	Inclusion	Exclusion
Population	Adult & young-adult patients diagnosed with sarcoidosis	Patients diagnosed with other granulomatous diseases
Exposure	Sarcoidosis presenting in the oral cavity	Sarcoidosis in other regions of the body
Comparison	N/A	N/A
Outcome	Clinical features Diagnostic methods Treatment and outcome	Incomplete demographic, histopathological, and clinical details.
Study design	Case reports & case series Full-text availability English language only	Review articles, editorials, animal studies and conference abstracts Non-English language articles Duplicates that may introduce bias

**Table 2:** Comparison between Sarcoidosis with and without Bone Involvement.

Clinical Characteristics	With Bone Involvement (*n* = 26)		Without Bone Involvement (*n* = 51)		
Average age	42.3		44.3		
M:F ratio	5:8		11:25		
Duration	1 mos–3 yrs		4 days–10 yrs		
Symptoms *	*n*	%	*n*	%	*p* value
Swelling	4	15.4	19	38	0.040
Nodule	4	15.4	18	36	0.056
Bone loss	18	69.2	5	10	<0.001
Asymptomatic	4	15.4	5	10	0.355
Ulcerations	2	7.7	11	22	0.109
Erythema	4	15.4	7	14	0.547
Pain	7	26.9	6	12	0.089
Painless	5	19.2	9	18	0.547
Discomfort	1	3.8	2	4	0.738
Mobile teeth	9	34.6	1	2	<0.001
Gingival recession	3	11.5	1	2	0.109
Periodontitis	1	3.8	1	2	0.564
Papular lesion	1	3.8	2	4	0.738
Nasal obstruction	4	15.4	0	0	0.011
Gingival hyperplasia	0	0	6	12	0.076
Asymmetry	0	0	3	6	0.285
Failing implants	2	7.7	0	0	0.111
Bleeding	0	0	7	14	0.048
Reduction in tongue mobility	0	0	2	4	0.436
Location					
Mandible	12	46.2	20	39.2	0.116
Maxilla	10	38.5	12	23.5	
Generalized	4	15.4	19	37.3	
Diagnosis					
New	12	46.2	36	70.6	0.033
Pre-existing	14	53.8	15	29.4	
Treatment					
Combination	9	34.6	9	17.6	0.080
Surgical	7	26.9	6	11.8	
Steroids	5	19.2	14	27.5	
Non-surgical	2	7.7	12	23.5	
None	1	3.8	8	15.7	
N/A	2	7.7	2	3.9	
Outcome					
Resolved	20	76.9	36	70.6	0.218
Worsened	1	3.8	0	0	
Spontaneous remission	0	0	2	3.9	
No change	0	0	5	9.8	
N/A	5	19.2	8	15.7	

* One case presented multiple symptoms, hence total *n* for symptoms may not align with total *n* for each group.

## Data Availability

The original contributions presented in this study are included in the article. Further inquiries can be directed to the corresponding author(s).

## Supplementary Materials

The following supporting information can be downloaded at: https://www.mdpi.com/article/10.3390/jcm14197006/s1, Table S1. Study characteristics of cases with bone involvement. Table S2. Study characteristics of cases without bone involvement. Table S3. Studies quality assessment.
