# Supplementary material for: Clinical and Demographic Characteristics of Oral Sarcoidosis: A Systematic Review of Case Reports and Case Series
Source: J Clin Med. 2025 Oct 3;14(19):7006. doi: 10.3390/jcm14197006 (PMC12524915; doi:10.3390/jcm14197006)
Supplement: Supplementary file 1 [file jcm-14-07006-s001.zip › jcm-3861837-supplementary.pdf]

Table S1. Study characteristics of cases with bone involvement.

Author/year	Country	Age (yrs)	Sex	Initial symptoms	Time to presentation	Location	Diagnosis of sarcoidosis	Treatment	Outcome
**Poe 1943** [8]	US	41	F	Left ear pain	2 yrs	Posterior mandible	New	-	-
**Van Swol 1973** [9]	US	22	F	Tooth mobility, pain, periodontal disease, & gingival recession	3 yrs	Generalized	Pre-existing	Non-surgical intervention	Resolved
**Hillerup 1976** [10]	Denmark	22	F	Asymptomatic radiolucent lesion, small ulceration, bone loss	1 yr	Posterior mandible	Pre-existing	None	Resolved
**Betten & Koppang 1976** [11]	Norway	40	M	Asymptomatic bone loss	-	Posterior mandible	Pre-existing	Surgical intervention	Resolved
**Schwartz 1981** [12]	US	29	F	Right TMJ pain	1 yr	Posterior mandible	Pre-existing	Surgical intervention	Resolved
**Cohen & Reinhardt 1982** [13]	US	59	F	Non-healing socket, pain & paraesthesia.	6 mos	Posterior mandible	Pre-existing	Surgical intervention	Recurred
**Verheijen-Breemhaar et al. 1987** [14]	The Netherland s	28	F	Bone loss & loose teeth, nasal obstruction	1 mo	Anterior maxilla	Pre-existing	Combination	-
**Hildebrand et al. 1990** [15]	US	41	M	Nodule - lower lip, non-tender papular lesion - hard palate. Nasal obstruction, bone loss & loose teeth	6 mos	Anterior maxilla	Pre-existing	Steroids	Resolved
**Rubin et al. 1991** [16]	US	25	F	Bone loss<br>Multiple nodular lesions<br>Nasal obstruction	-	Anterior maxilla	Pre-existing	Combination	-
**Clayman et al. 1998** [17]	US	33	F	Painless swelling of the palate, bone loss & loose teeth	2 mos	Anterior maxilla	New	Combination	Worsened
**Hong & Farish 2000** [18]	US	40	M	Bone loss & loose teeth, discomfort	2 mos	Anterior maxilla	Pre-existing	Combination	Resolved
**White & Crocker 2000** [19]	US	37	M	Maxillary bone loss	2 yrs	Anterior maxilla	New	Surgical intervention	Resolved
**Suresh et al. 2004** [20]	US	46	M	Bone loss & loose teeth, gingival recession	3 yrs	Posterior mandible	Pre-existing	Steroids + TNF receptor antagonist	Resolved

**Suresh et al. 2004** [21]	US	25	F	Painless enlarging erythematous swelling & redness of the roof of the mouth	3 mos	Maxilla (Palate)	New	Steroids	Resolved
**Grimaldi et al. 2004** [22]	Italy	33	F	Fistula in the buccal sulcus near lower left canine<br>Bone loss	3 mos	Mandible	New	Surgical intervention	Resolved
**Moretti et al. 2007** [23]	US	47	M	Severe pain in the left mandible<br>Erythematous and oedematous gingival tissues with recession<br>Nodule on the floor of the mouth<br>Bone loss	2 yrs	Posterior mandible	Pre-existing	Combination	Resolved
**Wright & Ambro 2009** [24]	US	52	F	Nasal obstruction, friable mucosa with nodules, non-tender erythema of hard palate<br>Osteolytic lesions	-	Premaxilla	Pre-existing	Steroids	-
**Cain et al. 2012** [25]	US	43	M	Dysgeusia<br>Mandibular osteolyitc lesion	-	Mandible	New	Surgical intervention	Resolved
**Wiseli et al. 2015** [26]	Switzerlan d	74	F	Pain in the right lower jaw<br>Bone loss	Months	Mandible	New	Immunosuppress ive therapy	Resolved
**Gupta et al. 2015** [27]	India	40	M	Ulcerated asymptomatic lesions<br>Erythematous gingival lesion<br>Multiple mobile teeth<br>Osteolytic lesion	2-3 yrs	Generalized	New	Non-surgical treatment + steroids	Resolved
**Hori et al. 2020** [28]	Tokyo	51	F	Right-side TMJ pain & trismus	3 mos	Mandible	Pre-existing	Steroids	Resolved
**Hosni et al. 2021** [29]	UK	65	F	Asymptomatic non-healing dental socket (oroantral fistula)	4 mos	Generalized	New	Combination	Resolved
**Cheema et al. 2022** [30]	US	57	F	Peri-implantitis	2 yrs	Mandible	New	Combination	Resolved
**Koutrakis et al. 2022** [31]	US	38	M	Failing mandibular implants<br>Bone loss & tooth mobility	3 yrs	Generalized	New	-	-
**Mittal et al. 2024**[32]	India	62	M	Progressively enlarging painless well-defined swelling, bone loss	3 yrs	Premaxilla	New	Excised	Resolved
**Patel et al. 2024**[33]	UK	50	F	Firm, discrete neck swelling<br>Bone loss & mobile anterior teeth	1 yr	Maxilla	Pre-existing	Steroids	Resolved

**Total**	9 countries	Avg: 42.3<br>Range:<br>22 - 74 yrs<br>SD: 13.7	Total - 26<br>M - 10<br>F - 16	Refer to Table 5	Range: 1- 36 mos<br>Avg: 16.1 mos	Mandible - 12<br>Maxilla - 8<br>Generalized – 4<br>Premaxilla - 2	New – 12<br>Pre-existing - 14	Steroids<br>Surgical intervention<br>Combination<br>None<br>N/A	Resolved – 19<br>Worsened - 2<br>N/A - 5

Table S2. Study characteristics of cases without bone involvement.

Author/year	Country	Age (yrs)	Sex	Symptoms	Location	Duration	Diagnosis of sarcoidosis	Treatment	Outcome
**Hoggins et al. 1969** [34]	UK	45	F	Firm swelling	Left cheek & maxilla	12 mos	New	Surgical excision + radiotherapy	Resolved
**Van Maarsseveen et al. 1982** [35]	The Netherlands	23	M	Multiple firm nodules	Hard & soft palate	Unknown	Pre-existing	None	No change
		69	F	Small, firm, mobile submucosal nodule	Tongue	Some mos	New	Surgical excision	Resolved
		25	M	Well-circumscribed submandibular swelling	Submandibular	6 mos	New	Surgical excision	Resolved
**Sloan et al. 1983** [36]	UK	16	M	Gingival hyperplasia, halitosis, & bleeding with minor trauma<br>Localized bone loss (upper anterior teeth)	Gingiva	-	New	Surgical excision + steroids + periodontal therapy	Resolved
**DeLuke & Sciubba 1985** [37]	US	35	F	Firm, non-tender, slowly enlarging left facial swelling	Buccal mucosa	4 wks	New	Steroids	Resolved
**Macleod et al. 1985** [38]	UK	50	F	Slowly increasing swelling & indurations	Tongue	3 yr	New	Steroids	Resolved
**Hildebrand et al. 1990** [15]	US	36	M	Papular lesions	Hard palate	-	Pre-existing	Steroids	Resolved
**Mendelsohn et al. 1992** [39]	UK	43	M	Two symmetrical indurated swellings & reduction in tongue mobility	Tongue	8 mos	New	None	No change
**Soto et al. 1997** [40]	Spain	56	F	Asymptomatic papular lesion on the ventral surface of the tongue, slowly enlarging. And submucosal nodule in the buccal submucosa.	Tongue	5 mos	Pre-existing	Steroids	Improved
**Piattelli et al. 1998** [41]	UK	54	M	Ulcerated, non-tender, indurated swelling	Lower lips	2 mos	New	-	-
**Nagata et al. 1999** [42]	Japan	32	F	Asymptomatic nodule	Tongue	1 mo	New	None	No change
**Ho & Blair 2003** [43]	UK	58	F	Localised area of redness & swelling<br>Occasional gum bleeding	Gingiva	18 mos	Pre-existing	Non-surgical treatment	Resolved

				Mild bone loss					
**Armstrong et al. 2004** [44]	Ireland	39	F	Painful ulcerated gingiva & lingual swelling	Gingiva	2 mos	New	Non-surgical treatment	Spontaneous remission
**Kasamatsu et al. 2007** [45]	Japan	71	F	Painless buccal nodule, facial asymmetry	Buccal mucosa	2 wks	Pre-existing	None	Spontaneous remission
**Koike et al. 2007** [46]	Japan	48	M	Firm submucosal swelling & indurations, reduced mobility with no pain	Tongue	2 mos	New	Steroids	Resolved
**Poate et al. 2008**[47]	UK	33	F	Asymptomatic swelling	Tongue	3 mos	New	Steroids	Resolved
		41	F	Generalized swelling of the gingiva with areas of erythema	Gingiva	-	New	No treatment	-
		43	F	Nodule on the upper lip & palate, discomfort with eating.	Lips	1 yr	Pre-existing	Steroids	-
**Antunes et al. 2008** [48]	Brazil	57	F	Painful erythematous erosive/ulcerated macule & spontaneous bleeding<br>Mild bone loss	Maxillary gingiva	3 yrs	Pre-existing	Non-surgical treatment + steroids	Resolved
**Kolokotronis et al. 2009** [49]	Greece	46	F	Nodular lesions on lower lip & hard palate, erythematous & hyperplastic gingiva	Hard palate<br>Lower lips<br>Gingiva	6 yrs	New	-	-
**Bouaziz et al. 2012**[50]	France	Avg: 38	9 F<br>3 M	Nodules – 7<br>Ulcers – 5	Tongue – 4<br>Lips – 3<br>Oral mucosa – 2<br>Palate – 2<br>Gingiva - 1	Avg: 2.8 yrs	New – 7<br>Pre-existing - 5	No treatment - 3<br>Non-surgical treatment - 6<br>Surgical excision - 1<br>Combination - 2	Resolved – 10<br>No change – 2
**Kadiwala & Dixit 2013** [51]	India	36	F	Generalised overgrowth of gingiva, pain and occasional bleeding<br>Localized bone loss (upper & lower anterior)	Gingiva	10 yrs	New	Surgical excision	Resolved

**Motswaledi et al. 2014** [52]	South Africa	48	F	Asymptomatic nodular lesions on her lower labial mucosa & erythematous area with a granular surface on the buccal gingiva	Gingiva<br>Lower labial mucosa	-	New	Steroids	Resolved
**Tripathi et al. 2014** [53]	India	42	M	Intraoral localised painless swelling (maxillary anterior gingiva)	Gingiva	1 mo	New	Surgical excision + steroids	Resolved
**Kalsi et al. 2016** [54]	UK	39	F	Swollen lower lip & spontaneous gingival bleeding, nodular areas with soft tissue hyperplasia in the lower labial gingiva.	Gingiva<br>Buccal mucosa	4 days	New	Platelet level correction	Resolved
**Radochova et al. 2016** [55]	Czech Republic	59	F	Painless aphthous (erosive) lesions<br>Submucosal palpable mass	Lower lip<br>Buccal mucosa	3 mos	Pre-existing	Steroids	Resolved
**Gill & Siddiqi 2017** [56]	UK	39	F	Painless swelling in hard palate.	Hard palate	4 wks	New	Steroids	Resolved
**Carey et al. 2019** [57]	UK	64	F	Submucosal tender mass on the palate	Hard palate	2 wk	Pre-existing	Steroids	-
**Bagchi et al. 2019** [58]	India	48	F	Swelling and discomfort in the left cheek region	Buccal mucosa	1 yr	New	Steroids	Resolved
**Gulseren & Elcin 2020** [59]	Turkey	31	M	Yellow to pink coloured lesions on his lower labial mucosa	Lower labial mucosa	3 mos	New	Oral hydroxychloroquine	Resolved
**Koutrakis et al. 2022** [31]	US	50	F	Gingival erythema and ulcerations, increasing tooth mobility.	Gingiva	-	New	TNF injections	-
		59	F	Non-tender indurated lip lesion	Lower lips	5 yrs	Pre-existing	Steroids	-
**Shahabinejad et al. 2023** [60]	Iran	47	F	Red, non-tender diffuse swelling of the lips & asymmetry + lymphadenopathy<br>Multi-lobular, rubbery, exophytic lesion in the buccal mucosa.	Upper lips<br>Buccal mucosa	5 mos	New	Steroids	Resolved
**Galohda & Shreehari 2023** [61]	India	38	F	Enlargement of gums, difficulty eating and brushing, and occasional bleeding<br>Persistent upper lip swelling<br>Localized bone loss (upper & lower anterior)	Gingiva<br>Upper & lower lip	1 yr	New	Surgical excision + steroids	Resolved

**Khongsit et al. 2023** [62]	India	25	F	Swelling on right cheek with ulcerations & pain with swallowing<br>Mild facial asymmetry & lymphadenopathy	Retromolar trigone region	2 mos	New	Steroids	Resolved
**Borges et al. 2023**[63]	Brazil	85	F	Persistently growing asymptomatic nodule on the upper lip	Upper lip	3 mos	New	Excised	Resolved
**Medeiros et al. 2024**[64]	Brazil	47	M	Isolated submucosal nodule in the midline of the lower lip	Lower lip	3 mos	New	Excised	Resolved
**Venugopalan et al. 2024** [65]	India	14	F	Burning sensation in her gums, & overgrown with bleeding and pain	Gingiva	10 mos	New	Non-surgical treatment+ steroids + hydroxychloroquine	Resolved
**Swain et al. 2024** [66]	US	43	M	Severe periodontitis with severe gum recession	Gingiva	2 yrs	New	Non-surgical intervention	-
**Total**	14 countries	Avg: 44.3<br>Range:<br>14 - 85<br>SD: 14.6	Total - 51<br>M - 14<br>F - 37	Refer to Table 5	Gingiva – 15<br>Lip - 15<br>Tongue - 11<br>Buccal mucosa – 8<br>Palate - 7<br>Submandibular - 1<br>Retromolar trigone - 1	Range:<br>4 days - 10 yrs<br>Avg; 15 mos	New - 36<br>Pre-existing - 15	Steroids<br>Surgical excision<br>Non-surgical treatment<br>Combination<br>None<br>N/A	Resolved - 36<br>No change - 5<br>Spontaneous remission - 2<br>N/A - 8

Table S3. Studies quality assessment

Author/year	Demographic	History	Current clinical condition	Diagnostic tests	Treatment described	Post-Intervention Clinical Condition	Adverse events	Takeaway Lessons	Overall appraisal:
**Poe 1943** [16]	X	X	X	X				X	5
**Hoggins et al. 1969** [42]	X	X	X	X	X	X	X	X	8
**Van Swol 1973** [17]	X	X	X	X	X	X		X	7
**Hillerup 1976** [18]	X	X	X	X		X		X	6
**Betten & Koppang 1976** [19]	X	X	X	X	X	X		X	7
**Schwartz 1981** [20]	X		X	X	X	X		X	6
**Van Maarsseveen et al. 1982** [43]	X		X	X	X	X		X	6
**Cohen & Reinhardt 1982** [21]	X	X	X	X	X		X	X	7
**Sloan et al. 1983** [44]	X		X	X	X	X	X	X	7
**DeLuke & Sciubba 1985** [45]	X	X	X	X	X	X		X	7
**Macleod et al. 1985** [46]	X	X	X	X	X	X		X	8
**Verheijen-Breemhaar et al.**	X	X	X	X	X	X	X	X	8
**Hildebrand et al. 1990** [23]	X	X	X	X	X	X	X		7
**Rubin et al. 1991** [24]	X	X	X	X	X	X		X	7
**Mendelsohn et al. 1992** [47]	X		X	X	X	X		X	6
**Soto et al. 1997** [48]	X	X	X	X	X	X		X	7
**Clayman et al. 1998** [25]	X	X	X	X	X	X	X	X	8
**Piattelli et al. 1998** [49]	X		X	X				X	4
**Nagata et al. 1999** [50]	X		X	X	X	X	X	X	7
**Hong & Farish 2000** [26]	X	X	X	X	X	X		X	7
**White & Crocker 2000** [27]	X		X	X	X	X		X	6

**Ho & Blair 2003** [51]	X	X	X	X	X	X	X	X	8
**Suresh et al. 2004** [28]	X	X	X	X	X	X		X	7
**Suresh et al. 2004** [29]	X	X	X	X	X			X	6
**Grimaldi et al. 2004** [30]	X		X	X		X		X	5
**Armstrong et al. 2004** [52]	X	X	X	X	X	X		X	6
**Moretti et al. 2007** [31]	X	X	X	X	X	X	X	X	8
**Kasamatsu et al. 2007** [53]	X	X	X	X	X	X		X	7
**Koike et al. 2007** [54]	X		X	X	X	X		X	6
**Poates et al. 2008**[55]	X	X	X	X	X			X	6
**Antunes et al. 2008** [56]	X	X	X	X	X	X	X	X	8
**Kolokotronis et al. 2009** [57]	X	X	X	X				X	5
**Wright & Amber 2009** [32]	X		X	X	X			X	5
**Bouaziz et al. 2012**[58]	X	X	X	X	X	X	X	X	8
**Cain et al. 2012** [33]	X	X	X	X	X	X	X	X	8
**Kadiwala & Dixit 2013** [59]	X	X	X	X	X	X		X	7
**Motswaledi et al. 2014** [60]	X		X	X	X	X		X	6
**Tripathi et al. 2014** [61]	X	X	X	X	X	X		X	7
**Wiseli et al. 2015** [34]	X	X	X	X	X	X		X	7
**Gupta et al. 2015** [35]	X	X	X	X	X	X		X	7
**Kalsi 2016** [62]	X		X	X	X	X		X	6
**Radochova et al. 2016** [63]	X	X	X	X	X	X		X	7
**Gill & Siddiqi**	X	X	X	X	X	X		X	7
**Carey et al. 2019** [65]	X	X	X	X	X		X	X	8

**Bagchi 2019** [66]	X	X	X	X	X	X		X	7
**Gulseren & Elcin 2020** [67]	X	X	X	X	X	X		X	7
**Hori et al. 2020** [36]	X	X	X	X	X	X		X	7
**Hosni et al. 2021** [37]	X	X	X	X	X	X	X	X	8
**Cheema et al. 2022** [38]	X	X	X	X	X	X	X	X	8
**Koutrakis et al. 2022** [39]	X	X	X	X				X	5
**Shahabinejad et al. 2023** [68]	X	X	X	X	X	X	X	X	8
**Galohda & AK 2023** [69]	X	X	X	X	X	X		X	7
**Khongsit et al. 2023** [70]	X	X	X	X	X	X		X	6
**Borges et al. 2023**[71]	X	X	X	X	X	X		X	7
**Medeiros et al. 2024**[72]	X	X	X	X	X	X		X	7
**Venugopalan et al. 2024** [73]	X		X	X	X			X	5
**Swain et al. 2024** [74]	X	X	X	X	X			X	6
**Mittal et al. 2024**[40]	X	X	X	X	X	X		X	7
**Patel et al. 2024**[41]	X	X	X	X	X	X		X	7

groups * Locations Crosstabulation

			Locations			Total
			Mandible	Maxilla	Generalized	
groups	Bone involvement	Count	12	10	4	26
		% within groups	46.2%	38.5%	15.4%	100.0%
		% within Locations	37.5%	45.5%	17.4%	33.8%
		% of Total	15.6%	13.0%	5.2%	33.8%
	Non-bone involvement	Count	20	12	19	51
		% within groups	39.2%	23.5%	37.3%	100.0%
		% within Locations	62.5%	54.5%	82.6%	66.2%
		% of Total	26.0%	15.6%	24.7%	66.2%
Total		Count	32	22	23	77
		% within groups	41.6%	28.6%	29.9%	100.0%
		% within Locations	100.0%	100.0%	100.0%	100.0%
		% of Total	41.6%	28.6%	29.9%	100.0%

Chi-Square Tests

	Value	df	Asymptotic Significance (2-sided)
Pearson Chi-Square	4.301[a]	2	.116
Likelihood Ratio	4.569	2	.102
Linear-by-Linear Association	2.013	1	.156
N of Valid Cases	77		

a. 0 cells (0.0%) have expected count less than 5. The minimum expected count is 7.43.

groups * Dx Crosstabulation

			Dx		
			New	Pre-existing	Total
groups	Bone involvement	Count	12	14	26
		% within groups	46.2%	53.8%	100.0%
		% within Dx	25.0%	48.3%	33.8%
		% of Total	15.6%	18.2%	33.8%
	Non-bone involvement	Count	36	15	51
		% within groups	70.6%	29.4%	100.0%
		% within Dx	75.0%	51.7%	66.2%
		% of Total	46.8%	19.5%	66.2%
Total		Count	48	29	77
		% within groups	62.3%	37.7%	100.0%
		% within Dx	100.0%	100.0%	100.0%
		% of Total	62.3%	37.7%	100.0%

Chi-Square Tests

	Value	df	Asymptotic Significance (2-sided)	Exact Sig. (2-sided)	Exact Sig. (1-sided)
Pearson Chi-Square	4.379[a]	1	.036		
Continuity Correction[b]	3.400	1	.065		
Likelihood Ratio	4.327	1	.038		
Fisher's Exact Test				.048	.033
Linear-by-Linear Association	4.322	1	.038		
N of Valid Cases	77				

a. 0 cells (0.0%) have expected count less than 5. The minimum expected count is 9.79.

b. Computed only for a 2x2 table

groups * Rx Crosstabulation

			Rx						Total
			Combination	Surgical Treatment	Steriods	Non-Surgical treatment	No treatment	N/A	
groups	Bone involvement	Count	9	7	5	2	1	2	26
		% within groups	34.6%	26.9%	19.2%	7.7%	3.8%	7.7%	100.0%
		% within Rx	50.0%	53.8%	26.3%	14.3%	11.1%	50.0%	33.8%
		% of Total	11.7%	9.1%	6.5%	2.6%	1.3%	2.6%	33.8%
	Non-bone involvement	Count	9	6	14	12	8	2	51
		% within groups	17.6%	11.8%	27.5%	23.5%	15.7%	3.9%	100.0%
		% within Rx	50.0%	46.2%	73.7%	85.7%	88.9%	50.0%	66.2%
		% of Total	11.7%	7.8%	18.2%	15.6%	10.4%	2.6%	66.2%
Total		Count	18	13	19	14	9	4	77
		% within groups	23.4%	16.9%	24.7%	18.2%	11.7%	5.2%	100.0%
		% within Rx	100.0%	100.0%	100.0%	100.0%	100.0%	100.0%	100.0%
		% of Total	23.4%	16.9%	24.7%	18.2%	11.7%	5.2%	100.0%

Chi-Square Tests

	Value	df	Asymptotic Significance (2-sided)
Pearson Chi-Square	9.849[a]	5	.080
Likelihood Ratio	10.373	5	.065
Linear-by-Linear Association	4.582	1	.032
N of Valid Cases	77		

a. 5 cells (41.7%) have expected count less than 5. The minimum expected count is 1.35.

groups * outcomes Crosstabulation

			outcomes					Total
			Resolved	Worsened	Spontaneous remission	No change	N/A	
groups	Bone involvement	Count	19	2	0	0	5	26
		% within groups	73.1%	7.7%	0.0%	0.0%	19.2%	100.0%
		% within outcomes	34.5%	100.0%	0.0%	0.0%	38.5%	33.8%
		% of Total	24.7%	2.6%	0.0%	0.0%	6.5%	33.8%
	Non-bone involvement	Count	36	0	2	5	8	51
		% within groups	70.6%	0.0%	3.9%	9.8%	15.7%	100.0%
		% within outcomes	65.5%	0.0%	100.0%	100.0%	61.5%	66.2%
		% of Total	46.8%	0.0%	2.6%	6.5%	10.4%	66.2%
Total		Count	55	2	2	5	13	77
		% within groups	71.4%	2.6%	2.6%	6.5%	16.9%	100.0%
		% within outcomes	100.0%	100.0%	100.0%	100.0%	100.0%	100.0%
		% of Total	71.4%	2.6%	2.6%	6.5%	16.9%	100.0%

Chi-Square Tests

	Value	df	Asymptotic Significance (2-sided)
Pearson Chi-Square	7.635[a]	4	.106
Likelihood Ratio	10.251	4	.036
Linear-by-Linear Association	.160	1	.689
N of Valid Cases	77		

a. 7 cells (70.0%) have expected count less than 5. The minimum expected count is .68.

groups * Swelling

Crosstab

		Swelling				Total	
		Absent		Present			
		N	%	N	%	N	%
groups	Bone involvement	22	40.7%	4	17.4%	26	33.8%
	Non-bone involvement	32	59.3%	19	82.6%	51	66.2%
Total		54	100.0%	23	100.0%	77	100.0%

Chi-Square Tests

	Value	df	Asymptotic Significance (2-sided)	Exact Sig. (2-sided)	Exact Sig. (1-sided)
Pearson Chi-Square	3.932[a]	1	.047		
Continuity Correction[b]	2.957	1	.085		
Likelihood Ratio	4.228	1	.040		
Fisher's Exact Test				.066	.040
Linear-by-Linear Association	3.881	1	.049		
N of Valid Cases	77				

a. 0 cells (0.0%) have expected count less than 5. The minimum expected count is 7.77.

b. Computed only for a 2x2 table

groups * Nodule

Crosstab

		Nodule					
		Absent		Present		Total	
		N	%	N	%	N	%
groups	Bone involvement	22	40.0%	4	18.2%	26	33.8%
	Non-bone involvement	33	60.0%	18	81.8%	51	66.2%
Total		55	100.0%	22	100.0%	77	100.0%

Chi-Square Tests

	Value	df	Asymptotic Significance (2-sided)	Exact Sig. (2-sided)	Exact Sig. (1-sided)
Pearson Chi-Square	3.345[a]	1	.067		
Continuity Correction[b]	2.440	1	.118		
Likelihood Ratio	3.585	1	.058		
Fisher's Exact Test				.108	.056
Linear-by-Linear Association	3.301	1	.069		
N of Valid Cases	77				

a. 0 cells (0.0%) have expected count less than 5. The minimum expected count is 7.43.

b. Computed only for a 2x2 table

groups * Bone loss

Crosstab

		Bone loss					
		Absent		Present		Total	
		N	%	N	%	N	%
groups	Bone involvement	8	14.8%	18	78.3%	26	33.8%
	Non-bone involvement	46	85.2%	5	21.7%	51	66.2%
Total		54	100.0%	23	100.0%	77	100.0%

Chi-Square Tests

	Value	df	Asymptotic Significance (2-sided)	Exact Sig. (2-sided)	Exact Sig. (1-sided)
Pearson Chi-Square	29.032[a]	1	<.001		
Continuity Correction[b]	26.264	1	<.001		
Likelihood Ratio	29.090	1	<.001		
Fisher's Exact Test				<.001	<.001
Linear-by-Linear Association	28.655	1	<.001		
N of Valid Cases	77				

a. 0 cells (0.0%) have expected count less than 5. The minimum expected count is 7.77.

b. Computed only for a 2x2 table

groups * Asymptomatic

Crosstab

		Asymptomatic				Total	
		Absent		Present			
		N	%	N	%	N	%
groups	Bone involvement	22	32.4%	4	44.4%	26	33.8%
	Non-bone involvement	46	67.6%	5	55.6%	51	66.2%
Total		68	100.0%	9	100.0%	77	100.0%

Chi-Square Tests

	Value	df	Asymptotic Significance (2-sided)	Exact Sig. (2-sided)	Exact Sig. (1-sided)
Pearson Chi-Square	.520[a]	1	.471		
Continuity Correction[b]	.120	1	.729		
Likelihood Ratio	.501	1	.479		
Fisher's Exact Test				.477	.355
Linear-by-Linear Association	.513	1	.474		
N of Valid Cases	77				

a. 1 cells (25.0%) have expected count less than 5. The minimum expected count is 3.04.

b. Computed only for a 2x2 table

groups * Ulcerations

Crosstab

		Ulcerations					
		Absent		Present		Total	
		N	%	N	%	N	%
groups	Bone involvement	24	37.5%	2	15.4%	26	33.8%
	Non-bone involvement	40	62.5%	11	84.6%	51	66.2%
Total		64	100.0%	13	100.0%	77	100.0%

Chi-Square Tests

	Value	df	Asymptotic Significance (2-sided)	Exact Sig. (2-sided)	Exact Sig. (1-sided)
Pearson Chi-Square	2.363[a]	1	.124		
Continuity Correction[b]	1.478	1	.224		
Likelihood Ratio	2.636	1	.104		
Fisher's Exact Test				.199	.109
Linear-by-Linear Association	2.332	1	.127		
N of Valid Cases	77				

a. 1 cells (25.0%) have expected count less than 5. The minimum expected count is 4.39.

b. Computed only for a 2x2 table

groups * Erythema

Crosstab

		Erythema					
		Absent		Present		Total	
		N	%	N	%	N	%
groups	Bone involvement	22	33.3%	4	36.4%	26	33.8%
	Non-bone involvement	44	66.7%	7	63.6%	51	66.2%
Total		66	100.0%	11	100.0%	77	100.0%

Chi-Square Tests

	Value	df	Asymptotic Significance (2-sided)	Exact Sig. (2-sided)	Exact Sig. (1-sided)
Pearson Chi-Square	.039[a]	1	.844		
Continuity Correction[b]	.000	1	1.000		
Likelihood Ratio	.038	1	.845		
Fisher's Exact Test				1.000	.547
Linear-by-Linear Association	.038	1	.845		
N of Valid Cases	77				

a. 1 cells (25.0%) have expected count less than 5. The minimum expected count is 3.71.

b. Computed only for a 2x2 table

groups * Pain

Crosstab

		Pain					
		Absent		Present		Total	
		N	%	N	%	N	%
groups	Bone involvement	19	29.7%	7	53.8%	26	33.8%
	Non-bone involvement	45	70.3%	6	46.2%	51	66.2%
Total		64	100.0%	13	100.0%	77	100.0%

Chi-Square Tests

	Value	df	Asymptotic Significance (2-sided)	Exact Sig. (2-sided)	Exact Sig. (1-sided)
Pearson Chi-Square	2.820[a]	1	.093		
Continuity Correction[b]	1.843	1	.175		
Likelihood Ratio	2.685	1	.101		
Fisher's Exact Test				.114	.089
Linear-by-Linear Association	2.783	1	.095		
N of Valid Cases	77				

a. 1 cells (25.0%) have expected count less than 5. The minimum expected count is 4.39.

b. Computed only for a 2x2 table

groups * Painless

Crosstab

		Painless				Total	
		Absent		Present			
		N	%	N	%	N	%
groups	Bone involvement	21	33.3%	5	35.7%	26	33.8%
	Non-bone involvement	42	66.7%	9	64.3%	51	66.2%
Total		63	100.0%	14	100.0%	77	100.0%

Chi-Square Tests

	Value	df	Asymptotic Significance (2-sided)	Exact Sig. (2-sided)	Exact Sig. (1-sided)
Pearson Chi-Square	.029[a]	1	.865		
Continuity Correction[b]	.000	1	1.000		
Likelihood Ratio	.029	1	.865		
Fisher's Exact Test				1.000	.547
Linear-by-Linear Association	.029	1	.866		
N of Valid Cases	77				

a. 1 cells (25.0%) have expected count less than 5. The minimum expected count is 4.73.

b. Computed only for a 2x2 table

groups * Discomfort

Crosstab

		Discomfort				Total	
		Absent		Present			
		N	%	N	%	N	%
groups	Bone involvement	25	33.8%	1	33.3%	26	33.8%
	Non-bone involvement	49	66.2%	2	66.7%	51	66.2%
Total		74	100.0%	3	100.0%	77	100.0%

Chi-Square Tests

	Value	df	Asymptotic Significance (2-sided)	Exact Sig. (2-sided)	Exact Sig. (1-sided)
Pearson Chi-Square	.000[a]	1	.987		
Continuity Correction[b]	.000	1	1.000		
Likelihood Ratio	.000	1	.987		
Fisher's Exact Test				1.000	.738
Linear-by-Linear Association	.000	1	.987		
N of Valid Cases	77				

a. 2 cells (50.0%) have expected count less than 5. The minimum expected count is 1.01.

b. Computed only for a 2x2 table

groups * Mobile teeth

Crosstab

		Mobile teeth					
		Absent		Present		Total	
		N	%	N	%	N	%
groups	Bone involvement	17	25.4%	9	90.0%	26	33.8%
	Non-bone involvement	50	74.6%	1	10.0%	51	66.2%
Total		67	100.0%	10	100.0%	77	100.0%

Chi-Square Tests

	Value	df	Asymptotic Significance (2-sided)	Exact Sig. (2-sided)	Exact Sig. (1-sided)
Pearson Chi-Square	16.250[a]	1	<.001		
Continuity Correction[b]	13.489	1	<.001		
Likelihood Ratio	16.080	1	<.001		
Fisher's Exact Test				<.001	<.001
Linear-by-Linear Association	16.039	1	<.001		
N of Valid Cases	77				

a. 1 cells (25.0%) have expected count less than 5. The minimum expected count is 3.38.

b. Computed only for a 2x2 table

groups * Gingival recession

Crosstab

		Gingival recession					
		Absent		Present		Total	
		N	%	N	%	N	%
groups	Bone involvement	23	31.5%	3	75.0%	26	33.8%
	Non-bone involvement	50	68.5%	1	25.0%	51	66.2%
Total		73	100.0%	4	100.0%	77	100.0%

Chi-Square Tests

	Value	df	Asymptotic Significance (2-sided)	Exact Sig. (2-sided)	Exact Sig. (1-sided)
Pearson Chi-Square	3.208[a]	1	.073		
Continuity Correction[b]	1.558	1	.212		
Likelihood Ratio	3.008	1	.083		
Fisher's Exact Test				.109	.109
Linear-by-Linear Association	3.166	1	.075		
N of Valid Cases	77				

a. 2 cells (50.0%) have expected count less than 5. The minimum expected count is 1.35.

b. Computed only for a 2x2 table

groups * Periodontitis

Crosstab

		Periodontitis					
		Absent		Present		Total	
		N	%	N	%	N	%
groups	Bone involvement	25	33.3%	1	50.0%	26	33.8%
	Non-bone involvement	50	66.7%	1	50.0%	51	66.2%
Total		75	100.0%	2	100.0%	77	100.0%

Chi-Square Tests

	Value	df	Asymptotic Significance (2-sided)	Exact Sig. (2-sided)	Exact Sig. (1-sided)
Pearson Chi-Square	.242[a]	1	.623		
Continuity Correction[b]	.000	1	1.000		
Likelihood Ratio	.229	1	.632		
Fisher's Exact Test				1.000	.564
Linear-by-Linear Association	.239	1	.625		
N of Valid Cases	77				

a. 2 cells (50.0%) have expected count less than 5. The minimum expected count is .68.

b. Computed only for a 2x2 table

groups * Papular lesion

Crosstab

		Papular lesion				Total	
		Absent		Present			
		N	%	N	%	N	%
groups	Bone involvement	25	33.8%	1	33.3%	26	33.8%
	Non-bone involvement	49	66.2%	2	66.7%	51	66.2%
Total		74	100.0%	3	100.0%	77	100.0%

Chi-Square Tests

	Value	df	Asymptotic Significance (2-sided)	Exact Sig. (2-sided)	Exact Sig. (1-sided)
Pearson Chi-Square	.000[a]	1	.987		
Continuity Correction[b]	.000	1	1.000		
Likelihood Ratio	.000	1	.987		
Fisher's Exact Test				1.000	.738
Linear-by-Linear Association	.000	1	.987		
N of Valid Cases	77				

a. 2 cells (50.0%) have expected count less than 5. The minimum expected count is 1.01.

b. Computed only for a 2x2 table

groups * Nasal obstruction

Crosstab

		Nasal obstruction				Total	
		Absent		Present			
		N	%	N	%	N	%
groups	Bone involvement	22	30.1%	4	100.0%	26	33.8%
	Non-bone involvement	51	69.9%	0	0.0%	51	66.2%
Total		73	100.0%	4	100.0%	77	100.0%

Chi-Square Tests

	Value	df	Asymptotic Significance (2-sided)	Exact Sig. (2-sided)	Exact Sig. (1-sided)
Pearson Chi-Square	8.276[a]	1	.004		
Continuity Correction[b]	5.447	1	.020		
Likelihood Ratio	9.124	1	.003		
Fisher's Exact Test				.011	.011
Linear-by-Linear Association	8.169	1	.004		
N of Valid Cases	77				

a. 2 cells (50.0%) have expected count less than 5. The minimum expected count is 1.35.

b. Computed only for a 2x2 table

groups * Fistula/Non-healing dental socket

Crosstab

		Fistula/Non-healing dental socket				Total	
		Absent		Present			
		N	%	N	%	N	%
groups	Bone involvement	23	31.1%	3	100.0%	26	33.8%
	Non-bone involvement	51	68.9%	0	0.0%	51	66.2%
Total		74	100.0%	3	100.0%	77	100.0%

Chi-Square Tests

	Value	df	Asymptotic Significance (2-sided)	Exact Sig. (2-sided)	Exact Sig. (1-sided)
Pearson Chi-Square	6.123[a]	1	.013		
Continuity Correction[b]	3.429	1	.064		
Likelihood Ratio	6.756	1	.009		
Fisher's Exact Test				.036	.036
Linear-by-Linear Association	6.044	1	.014		
N of Valid Cases	77				

a. 2 cells (50.0%) have expected count less than 5. The minimum expected count is 1.01.

b. Computed only for a 2x2 table

groups * Peri-implantitis

Crosstab

		Peri-implantitis				Total	
		Absent		Present			
		N	%	N	%	N	%
groups	Bone involvement	24	32.0%	2	100.0%	26	33.8%
	Non-bone involvement	51	68.0%	0	0.0%	51	66.2%
Total		75	100.0%	2	100.0%	77	100.0%

Chi-Square Tests

	Value	df	Asymptotic Significance (2-sided)	Exact Sig. (2-sided)	Exact Sig. (1-sided)
Pearson Chi-Square	4.028[a]	1	.045		
Continuity Correction[b]	1.561	1	.212		
Likelihood Ratio	4.448	1	.035		
Fisher's Exact Test				.111	.111
Linear-by-Linear Association	3.975	1	.046		
N of Valid Cases	77				

a. 2 cells (50.0%) have expected count less than 5. The minimum expected count is .68.

b. Computed only for a 2x2 table

groups * Bleeding

Crosstab

		Bleeding					
		Absent		Present		Total	
		N	%	N	%	N	%
groups	Bone involvement	26	37.1%	0	0.0%	26	33.8%
	Non-bone involvement	44	62.9%	7	100.0%	51	66.2%
Total		70	100.0%	7	100.0%	77	100.0%

Chi-Square Tests

	Value	df	Asymptotic Significance (2-sided)	Exact Sig. (2-sided)	Exact Sig. (1-sided)
Pearson Chi-Square	3.925[a]	1	.048		
Continuity Correction[b]	2.440	1	.118		
Likelihood Ratio	6.119	1	.013		
Fisher's Exact Test				.088	.048
Linear-by-Linear Association	3.875	1	.049		
N of Valid Cases	77				

a. 2 cells (50.0%) have expected count less than 5. The minimum expected count is 2.36.

b. Computed only for a 2x2 table

groups * Gingival hyperplasia

Crosstab

		Gingival hyperplasia					
		Absent		Present		Total	
		N	%	N	%	N	%
groups	Bone involvement	26	36.6%	0	0.0%	26	33.8%
	Non-bone involvement	45	63.4%	6	100.0%	51	66.2%
Total		71	100.0%	6	100.0%	77	100.0%

Chi-Square Tests

	Value	df	Asymptotic Significance (2-sided)	Exact Sig. (2-sided)	Exact Sig. (1-sided)
Pearson Chi-Square	3.317[a]	1	.069		
Continuity Correction[b]	1.882	1	.170		
Likelihood Ratio	5.199	1	.023		
Fisher's Exact Test				.091	.076
Linear-by-Linear Association	3.274	1	.070		
N of Valid Cases	77				

a. 2 cells (50.0%) have expected count less than 5. The minimum expected count is 2.03.

b. Computed only for a 2x2 table

groups * Asymmtery

Crosstab

		Asymmtery					
		Absent		Present		Total	
		N	%	N	%	N	%
groups	Bone involvement	26	35.1%	0	0.0%	26	33.8%
	Non-bone involvement	48	64.9%	3	100.0%	51	66.2%
Total		74	100.0%	3	100.0%	77	100.0%

Chi-Square Tests

	Value	df	Asymptotic Significance (2-sided)	Exact Sig. (2-sided)	Exact Sig. (1-sided)
Pearson Chi-Square	1.591[a]	1	.207		
Continuity Correction[b]	.408	1	.523		
Likelihood Ratio	2.533	1	.111		
Fisher's Exact Test				.547	.285
Linear-by-Linear Association	1.571	1	.210		
N of Valid Cases	77				

a. 2 cells (50.0%) have expected count less than 5. The minimum expected count is 1.01.

b. Computed only for a 2x2 table

groups * Reduction in tongue mobility

Crosstab

		Reduction in tongue mobility					
		Absent		Present		Total	
		N	%	N	%	N	%
groups	Bone involvement	26	34.7%	0	0.0%	26	33.8%
	Non-bone involvement	49	65.3%	2	100.0%	51	66.2%
Total		75	100.0%	2	100.0%	77	100.0%

Chi-Square Tests

	Value	df	Asymptotic Significance (2-sided)	Exact Sig. (2-sided)	Exact Sig. (1-sided)
Pearson Chi-Square	1.047[a]	1	.306		
Continuity Correction[b]	.071	1	.791		
Likelihood Ratio	1.675	1	.196		
Fisher's Exact Test				.547	.436
Linear-by-Linear Association	1.033	1	.309		
N of Valid Cases	77				

a. 2 cells (50.0%) have expected count less than 5. The minimum expected count is .68.

b. Computed only for a 2x2 table

groups * Parasthesia

Crosstab

		Parasthesia				Total	
		Absent		Present			
		N	%	N	%	N	%
groups	Bone involvement	25	32.9%	1	100.0%	26	33.8%
	Non-bone involvement	51	67.1%	0	0.0%	51	66.2%
Total		76	100.0%	1	100.0%	77	100.0%

Chi-Square Tests

	Value	df	Asymptotic Significance (2-sided)	Exact Sig. (2-sided)	Exact Sig. (1-sided)
Pearson Chi-Square	1.987[a]	1	.159		
Continuity Correction[b]	.119	1	.730		
Likelihood Ratio	2.197	1	.138		
Fisher's Exact Test				.338	.338
Linear-by-Linear Association	1.962	1	.161		
N of Valid Cases	77				

a. 2 cells (50.0%) have expected count less than 5. The minimum expected count is .34.

b. Computed only for a 2x2 table

Multiple Response Set

Custom Tables (All symptoms combined)

		groups					
		Bone involvement			Non-bone involvement		
		Count	Row N %	Column N %	Count	Row N %	Column N %
Symptoms	Swelling	4	17.4%	15.4%	19	82.6%	38.0%
	Nodule	4	18.2%	15.4%	18	81.8%	36.0%
	Bone loss	18	78.3%	69.2%	5	21.7%	10.0%
	Asymptomatic	4	44.4%	15.4%	5	55.6%	10.0%
	Ulcerations	2	15.4%	7.7%	11	84.6%	22.0%
	Erythema	4	36.4%	15.4%	7	63.6%	14.0%
	Pain	7	53.8%	26.9%	6	46.2%	12.0%
	Painless	5	35.7%	19.2%	9	64.3%	18.0%
	Discomfort	1	33.3%	3.8%	2	66.7%	4.0%
	Mobile teeth	9	90.0%	34.6%	1	10.0%	2.0%
	Gingival recession	3	75.0%	11.5%	1	25.0%	2.0%
	Periodontitis	1	50.0%	3.8%	1	50.0%	2.0%
	Papular lesion	1	33.3%	3.8%	2	66.7%	4.0%
	Nasal obstruction	4	100.0%	15.4%	0	0.0%	0.0%
	Fistula/Non-healing dental socket	3	100.0%	11.5%	0	0.0%	0.0%
	Peri-implantitis	2	100.0%	7.7%	0	0.0%	0.0%
	Bleeding	0	0.0%	0.0%	7	100.0%	14.0%
	Gingival hyperplasia	0	0.0%	0.0%	6	100.0%	12.0%
	Asymmtery	0	0.0%	0.0%	3	100.0%	6.0%
	Reduction in tongue mobility	0	0.0%	0.0%	2	100.0%	4.0%
	Parasthesia	1	100.0%	3.8%	0	0.0%	0.0%

Pearson Chi-Square Tests

		groups
$Symptoms	Chi-square	91.178
	df	21
	Sig.	<.001[*,b,c]

Results are based on nonempty rows and columns in each innermost subtable.

*. The Chi-square statistic is significant at the .05 level.

b. More than 20% of cells in this subtable have expected cell counts less than 5. Chi-square results may be invalid.

c. The minimum expected cell count in this subtable is less than one. Chi-square results may be invalid.
